# Diagnostic Utility of Plasma Biomarkers to Differentiate Controls, SCD, and MCI Subjects

**DOI:** 10.1002/alz.089935

**Published:** 2025-01-09

**Authors:** Allal Boutajangout, Arjun V. Masurkar, Ricardo S. Osorio, Wajiha Ahmed, Ludovic Debure, Alok Vedvyas, Jon Links, Karyn Marsh, Thomas Wisniewski

**Affiliations:** ^1^ Center for Cognitive Neurology, New York, NY USA; ^2^ NYU Grossman School of Medicine, New York, NY USA; ^3^ Center for Cognitive Neurology, New York University Langone Health, New York, NY USA; ^4^ 145 E 32nd St Fl 5, 145 E 32nd St Fl 5, NY USA

## Abstract

**Background:**

Plasma biomarkers of Alzheimer’s disease (AD) are less invasive and have lower costs than cerebrospinal fluid (CSF) biomarkers. Blood biomarkers are potential instruments for diagnosis, prognosis, and monitoring of disease progression. We assessed the diagnostic potential of several plasma biomarkers for the detection of early stages of AD.

**Method:**

152 participants (age>60) were recruited at the NYU Alzheimer's Disease Research Center. This cohort includes n=55 with normal cognition (NL), n=70 with subject cognitive decline (SCD), and n=27 with mild cognitive impairment (MCI). Comprehensive neuropsychological and magnetic resonance imaging evaluations were conducted for all patients. Plasma biomarkers assay Aβ40, Aβ42, total tau [t‐tau], neurofilament light [NfL], glial fibrillary acid protein [GFAP], ubiquitin carboxyl‐terminal hydrolase L1 [UCH‐L1], and pTau181 were measured using the SIMOA SR‐X a novel technology that employs highly sensitive immunoassays with a limit of detection (LOD) under 100 fg/ml.

**Result:**

The levels of Aβ40, Aβ42, and t‐tau were measured using the Neurology 3‐plex A. Aβ42 levels showed a significant difference between NL and MCI (one‐tailed t‐test p=0.0262). The level of Aβ42/Aβ40 ratio was also significantly different between SCD and MCI (one‐tailed t‐test p=0.0206). pTau181 level showed a statistically significant difference between NL and SCD (one‐tailed t‐test p=0.0341). The pTau181/Aβ42 ratio was significantly different between NL and SCD; and NL and MCI (one‐tailed t‐test p=0.0190 and p=0.0037, respectively). In addition, the t‐tau/Aβ42 ratio was significantly different between NL and SCD, and NL and MCI (p=0.0359 and p=0.0091, respectively). Correlation of these biomarkers with brain imaging and cognitive measures is underway.

**Conclusion:**

Biomarker levels of Aβ42, pTau181, pTau181/ Aβ42 ratio, and t‐tau/Aβ42 in plasma samples showed significant differences between the three groups of subjects. Plasma biomarkers can be useful for the diagnosis of AD‐related pathology at early stages.

